# Supplementary material for: Bioactivity and Chemical Profile of Rubus idaeus L. Leaves Steam-Distillation Extract
Source: Foods. 2022 May 17;11(10):1455. doi: 10.3390/foods11101455 (PMC9140405; doi:10.3390/foods11101455)
Supplement: Supplementary file 1 [file foods-11-01455-s001.zip › foods-1715186-supplementary.pdf]

foods

MDPI

Supplemental material

*Table S1. Bibliographic overview of R. idaeus extracts antioxidant activity. *=values unit normalised,* ***AA****=Ascorbic Acid,* ***CAE****=Chlorogenic Acid Equivalents,* ***DE****=Dry Extract,* ***DF****=Dry Fruit,* ***DM****=Dry Matter,* ***DW****=Dry Weight,* ***EC50****=Half Maximal Effective Concentration,* ***EtOH****=Ethanol,* ***$FeSO_4$****=Ferrous Sulfate,* ***FW****=Fresh Weight,* ***GA****=Gallic Acid,* ***GAE****=Gallic Acid Equivalents,* ***IC50****=Half Maximal Inhibitory Concentration,* ***MeOH****= Methanol,* ***NA****=Not Analysed,* ***TA****=Tannic Acid,* ***TE****=Trolox Equivalents.*

Cultivar	Method	TPC	DPPH	FRAP	ABTS
R. idaeus Berries [111]	Pulp crude extract Acidified MeOH (0.1% v/v HCl)	270±17.9 mg GAE/100g	588.9±5.5 µM TE/100g	1912.0±1.78 µM TE/100g FW	NA
	Juice crude extract Acidified MeOH (0.1% v/v HCl)	192.04±5.7 mg GAE/100g	363.0±3.4 µM TE/100g	623.0 ± 8.46 µM TE/100g FW	NA
R. idaeus var *September* Frozen berries [112]	MeOH/HCl 2% (95:5 v/v)	140.6±0.9 mg GAE/100g FW	EC50 10.9±0.2 mg	NA	NA
R. idaeus var *Summer* Frozen berries [112]		214.4±0.8 mg GAE/100g FW	EC50 5.5±0.5 mg	NA	NA
R. idaeus Air-dried leaves [95]	Acetone 70% + Trichloromethane	*26920±490 mg GAE/100g DE	*230±10 mM TE/100g DE	*216±4 mM TE/100g DE	*250±10 mM TE/100g DE
R. idaeus Fresh leaves [8]	MeOH crude exctract	*5968-9683 mg GAE/100g	IC50 110.17-199.18 µg/mL	NA	NA
R. idaeus Fresh berries [8]	MeOH crude exctract	*2429-3871 mg GAE/100g	IC50 246.09-489.60 µg/mL	NA	NA
R. idaeus Freeze-dried berries [113]	EtOH (96%) crude extract	*560-1370 mg GAE/100g; *130-330 mg GAE/100g DF	[50µL] 52,9-92,6%	NA	[50µL] 52,5-97,8%
R. idaeus var *Willamette* Dried berries [6]	Pomace from juice separation MeOH (80%) + acetic acid (0,05%)	*4370±202 mg GAE/100g	EC50 0.042±0.002 mg/mL	NA	NA
R. idaeus var *Meeker* Dried berries [6]		*2630±128 mg GAE/100g	EC50 0.072±0.004 mg/mL	NA	NA
R. idaeus Dried leaves [102]	EtOH (96%) crude extract	*480-1200 mg GAE/100g	20,5-82,5%	NA	8-42,7%
R. idaeus Dried leaves [114]	Infusion dH2O	*6890±560 mg GAE/100g DM	*10.52±1.29 g AA/100g DM	26.6±1.0 mM FeSO4/L	NA
R. idaeus var *Heritage* Lyophilized berries [9]	80% MeOH + 2% formic acid	196.55±14.9 mg GAE/100g FW	351.27 mg TE/100g FW	NA	NA
R. idaeus var *Summit* Lyophilized berries [9]		254.22±22.1 mg GAE/100g FW	351.27 mg TE/100g FW	NA	NA
R. idaeus var *Harvest*		179.41±27.3 mg GAE/100g FW	394.63 mg TE/100g FW	NA	NA

Lyophilized berries [9]					
R. idaeus var *Red Autumn Bliss* Lyophilized berries [9]		219.93±9.6 mg GAE/100g FW	476.20 mg TE/100g FW	NA	NA
R. idaeus var *Yellow Autumn Bliss* Lyophilized berries [9]		391.15±33.4 mg GAE/100g FW	351.27 mg TE/100g FW	NA	NA
R. idaeus Air-dried leaves [116]	EtOH (20%)	32.32±1.51 g% TA	EC50 0.099±0.008 mg/mL	EC50 0.068±0.003 mg/mL	EC50 0.227±0.027 mg/mL
R. idaeus var *Polana* Fresh berries [117]	EtOH (80%) crude maceration	*3664±80 mg GAE/100g DW	*29.4±2.1 mg TE/100g DW	NA	*25.5±1.9 mg TE/100g DW
R. idaeus var *Heritage* Berries [118]	EtOH + HCl 1.0N (85:15) maceration	974-7123 mg/100g DW	337-563 mg TE/100g FW	264-606 mg TE/100g FW	219-422 mg TE/100g FW
R. idaeus Dried berries [119]	Ethyl alcohol (50%) maceration	*2330 mg GAE/100g DW	IC50 64.03 μg/ml.	NA	NA
R. idaeus var *Tulameen* Frozen berries [120]	Acetone, water and acetic acid (70:29.5:0.5, v/v) maceration	*74870±2300 mg GAE/100g FW	NA	NA	*2250±40 mM TE/100g FW
R. idaeus var *Canby* Frozen berries [120]		*101240±3000 mg GAE/100g FW	NA	NA	*2040±20 mM TE/100g FW
R. idaeus var *Willamette* Frozen berries [120]		*76450±2000 mg GAE/100g FW	NA	NA	*2910±40 mM TE/100g FW
R. idaeus var *Newburgh* Frozen berries [120]		*124690±1600 mg GAE/100g FW	NA	NA	*3270±50 mM TE/100g FW
R. idaeus var *Heritage* Frozen berries [120]		*86440±1800 mg GAE/100g FW	NA	NA	*1940±10 mM TE/100g FW
R. idaeus var *Hollanda Boduru* Frozen berries [120]		*119010±3500 mg GAE/100g FW	NA	NA	*1790±10 mM TE/100g FW
R. idaeus var *Meeker* Frozen berries [120]		*112140±3000 mg GAE/100g FW	NA	NA	*3050±50 mM TE/100g FW
R. idaeus Freeze-dried berries [121]	MeOH (50%) Sonicated maceration	*4226±311 mg GAE/100g DW	IC50 18.71±1.35 μg/mL	*10081±1231 mM/100g DW	IC50 29.43±1.83 μg/mL
R. idaeus Freeze-dried leaves [121]		*6379±311 mg GAE/100g DW	IC50 5.60±0.31 μg/mL	*19832±2172 mM/100g DW	IC50 3.70±0.17 μg/mL

R. idaeus Freeze-dried seeds [121]		*2025±179 mg GAE/100g DW	IC50 32.33±1.42 µg/mL	*2192±372 mM/100g DW	IC50 33.15±2.19 µg/mL
R. idaeus Dried berries [122]	Water maceration	NA	IC50 92.62±0.47 µg/mL	NA	NA
	EtOH maceration	NA	IC50 94.10±0.20 µg/mL	NA	NA
	EtOH:water (1:1) maceration	NA	IC50 91.00±0.04 µg/mL	NA	NA
R. idaeus var *Oxycoccos* Fresh berries [103]	Water maceration	*624 mg GAE/100mL	[6.67%-33.5%] RSA 30-40%	NA	NA
R. idaeus var *Willamette* Fresh&frozen berries [123]	Pomace cold-press production, dried and n-hexane maceration	*17,8-20,4 mg GAE/100g	NA	NA	NA
R. idaeus Fresh berries [124]	Oil cold-press production and MeOH (80%) extraction	*16 mg GAE/100g oil	[40 mg/mL] RSA ~80%	NA	NA
	Oil cold-press production and MeOH (100%) extraction	*200 mg GAE/100g oil	[40 mg/mL] RSA ~20%		
R. idaeus 10 different genotypes Berries [94]	Acetone, water, and acetic acid (70:29.5:0.5) maceration	*114.5-203.1 mg GAE/100g FW	NA	*8.13-12.43 mM TE/100g FW	NA
R. idaeus var *Heritage* Berries [94]		*121.4 mg GAE/100g FW	NA	*8.37 mM TE/100g FW	NA
R. idaeus Berries [96]	Lyophilized and MeOH (50%) maceration	*1358±90 mg GAE/100g DW	NA	*16.59±0.04 mM TE/100g DW	*4.85-9.11 mM TE/100g DW
R. idaeus Leaves [96]	Oven-dried and MeOH (50%) infusion	*5045±940 mg GAE/100g DW	NA	*38.75±2.78 mM TE/100g DW	*68.5-126.97 mM TE/100g DW
R. idaeus var *Heritage* Frozen berries [93]	Acetone, water, and acetic acid (70:29.5:0.5) maceration	*180 mg GAE/100g FW	NA	*1810 µM TE/100g FW	*1750 µM TE/100g FW
R. idaeus var *Tulameen* Frozen berries [93]		*234 mg GAE/100g FW	NA	*1440 µM TE/100g FW	*1290 µM TE/100g FW
R. idaeus var *wild raspberry (14)* Frozen berries [93]		*0.149-0.348 mg GAE/100g FW	NA	*1120-1980 µM TE/100g FW	*890-2150 µM TE/100g FW
R. idaeus Dried berries [86]	*In vitro* gastric and small intestine digestion	~350-890 mg GAE/100g DW	*1.2-3.7 mM TE/100g FW	50-170 mM Fe(II)SE/g DW	*2-4 mM TE/100g FW
R. idaeus Dried seeds [86]		~100-770 mg GAE/100g DW	*0.5-3 mM TE/100g FW	30-80 mM Fe(II)SE/g DW	*700-2900 µM TE/100g FW
R. idaeus Dried leaves [101]	EtOH (96%) maceration	*30-220 mg GAE/100g DW	NA	NA	NA
R. idaeus var *Aksu Kırmızısı* Fresh berries	Water maceration	1040.9±15.9 mg GAE/100g FW	*7.66±0.026 mM TE/100g FW	NA	*6798±17 µM TE/100g FW

[87]	MeOH maceration	1787.3±12.5 mg GAE/100g FW	*12.24±0.33 mM TE/100g FW	NA	*8666±179 µM TE/100g FW
R. idaeus var *Rubin* Fresh berries [87]	Water maceration	1123.4±10.9 mg GAE/100g FW	*6.41±0.098 mM TE/100g FW	NA	*6436±173 µM TE/100g FW
	MeOH maceration	1866.2±7.6 mg GAE/100g FW	*9.66±0.44 mM TE/100g FW	NA	*7292±185 µM TE/100g FW
R. idaeus var *Newburgh* Fresh berries [87]	Water maceration	1355.8±16.1 mg GAE/100g FW	*8.37±0.11 mM TE/100g FW	NA	*8366±108 µM TE/100g FW
	MeOH maceration	1835.4±16.7 mg GAE/100g FW	*9.86±0.31 mM TE/100g FW	NA	*9856±307 µM TE/100g FW
R. idaeus var *Hollanda Boduru* Fresh berries [87]	Water maceration	1822.0±11.9 mg GAE/100g FW	*7.76±0.12 mM TE/100g FW	NA	*6970±103 µM TE/100g FW
	MeOH maceration	2062.3±4.1 mg GAE/100g FW	*12.76±0.18 mM TE/100g FW	NA	*11707±94 µM TE/100g FW
R. idaeus var *Heritage* Fresh berries [87]	Water maceration	1463.7±22.8 mg GAE/100g FW	*6.6±0.18 mM TE/100g FW	NA	*6544±27 µM TE/100g FW
	MeOH maceration	1924.7±19.6 mg GAE/100g FW	*8.12±0.15 mM TE/100g FW	NA	*7425±147 µM TE/100g FW
R. idaeus Berries [125]	Puree maceration with 20 minutes of sonication	*29.1-34.1 mg GAE/100mL	*352.2-746.4 µM/100mL	NA	NA
R. idaeus var *Willamette* Frozen berries [126]	Homogenisation	389.61±27.21 mg GAE/100g FW	278.01±7.66 µM TE/100g FW	NA	3.10±0.27 mM TE/100g FW
R. idaeus var *Meeker* Frozen berries [126]		415.39±95.52 mg GAE/100g FW	289.84±15.48 µM TE/100g FW	NA	3.37±0.63 mM TE/100g FW
R. idaeus Air-dried leaves [10]	EtOH (50%) infusion	6.24±0.51 g% GA	EC50 0.29±0.29 mg/mL	NA	NA
R. idaeus Berries [127]	Homogenisation, centrifugation and *in vitro* digestion	~3500 mg GAE/100g	NA	NA	NA
R. idaeus Fresh leaves [5]	MeOH (70%) + 0.1% HCl maceration, oven-dried, water infusion in ultrasound, diethyl ether/ethyl acetate (1:1) extraction	*230 mg GAE/100g DM	*350±30 µM TE/100g DM	716.0±5.1 µM Fe(II)SE/g DM	*100±3 µM TE/100g DM
	MeOH (70%) + 0.1% HCl maceration, oven-dried, MeOH infusion in ultrasound, diethyl ether/ethyl acetate (1:1) extraction	*540 mg GAE/100g DM	*870±50 µM TE/100g DM	3084.1±20.3 µM Fe(II)SE/g DM	*530±10 µM TE/100g DM
	MeOH (70%) + 0.1% HCl maceration, oven-dried, 2M HCl infusion in ultrasound, diethyl ether/ethyl acetate (1:1) extraction	*3171 mg GAE/100g DM	*4350±410 µM TE/100g DM	9675.2±15.2 µM Fe(II)SE/g DM	*3440±50 µM TE/100g DM
	MeOH (70%) + 0.1% HCl maceration, oven-dried, 2M NaOH infusion in ultrasound, diethyl ether/ethyl acetate (1:1) extraction	*~1600 mg GAE/100g DM	*1390 ±160 µM TE/100g DM	1994.2±17.5 µM Fe(II)SE/g DM	*1020±20 µM TE/100g DM
	MeOH (70%) + 0.1% HCl maceration, oven-dried, enzymatic hydrolysis, ultrasound infusion, diethyl ether/ethyl acetate (1:1) extraction	*~1750 mg GAE/100g DM	*1590±210 µM TE/100g DM	4299.8±31.5 µM Fe(II)SE/g DM	*2720±30 µM TE/100g DM
R. idaeus Fresh berries [5]	MeOH (70%) + 0.1% HCl maceration, oven-dried, water infusion in ultrasound, diethyl ether/ethyl acetate (1:1) extraction	*150 mg GAE/100g DM	*60±2 µM TE/100g DM	209.8±5.9 µM Fe(II)SE/g DM	*90±20 µM TE/100g DM
	MeOH (70%) + 0.1% HCl maceration, oven-dried, MeOH infusion in	*230 mg GAE/100g DM	*520±10 µM TE/100g DM	295.9±7.1 µM Fe(II)SE/g DM	*270±10 µM TE/100g DM

	ultrasound, diethyl ether/ethyl acetate (1:1) extraction				
	MeOH (70%) + 0.1% HCl maceration, oven-dried, 2M HCl infusion in ultrasound, diethyl ether/ethyl acetate (1:1) extraction	*902 mg GAE/100g DM	*3330±270 μM TE/100g DM	1138.9±11.2 μM Fe(II)SE/g DM	*2630±130 μM TE/100g DM
	MeOH (70%) + 0.1% HCl maceration, oven-dried, 2M NaOH infusion in ultrasound, diethyl ether/ethyl acetate (1:1) extraction	*~250 mg GAE/100g DM	*9600±90 μM TE/100g DM	164.3±14.7 μM Fe(II)SE/g DM	*4700±20 μM TE/100g DM
	MeOH (70%) + 0.1% HCl maceration, oven-dried, enzymatic hydrolysis, ultrasound infusion, diethyl ether/ethyl acetate (1:1) extraction	*~500 mg GAE/100g DM	*1200±180 μM TE/100g DM	322.4±23.3 μM Fe(II)SE/g DM	*1120±120 μM TE/100g DM
R. idaeus var *Yavuzlar(wild)* Berries [104]	Water maceration	*583±112 mg GAE/100g	0.90±0.12 μg TE	0.51±0.04 μg TE	1.01±0.13 μg TE
R. idaeus var *Yayla(wild)* Berries [104]		*2666±326 mg GAE/100g	0.90±0.05 μg TE	0.66±0.06 μg TE	0.93±0.12 μg TE
R. idaeus var *Yedigöl(wild)* Berries [104]		*1751±286 mg GAE/100g	0.62±0.03 μg TE	0.60±0.05 μg TE	0.94±0.10 μg TE
R. idaeus var *Willamette* Berries [104]		*1584±311 mg GAE/100g	0.63±0.10 μg TE	0.44±0.06 μg TE	0.98±0.07 μg TE
R. idaeus Fresh berries [97]	EtOH (60%) maceration	*~1300 mg GAE/100g DM	IC50 216.50±0.39 μg/mL	*2879±43 μM TE/100g DW	NA
R. idaeus Air-dried leaves [97]		*~2700 mg GAE/100g DM	IC50 68.10±0.74 μg/mL	*1393±20 μM TE/100g DW	NA
R. idaeus Pomace [92]	Water infusion	*158-215 mg GAE/100g	*53-89 mM TE/100g	*3840-4199 mM TE/100g	*1321-2159 mM TE/100g
	Water infusion with ultrasound	*199-253 mg GAE/100g	*65-98 mM TE/100g	*4772-5040 mM TE/100g	*1766-2333 mM TE/100g
R. idaeus Berries [128]	Microencapsulation by spray drying with EtOH (30%)	NA	20,61-41,52 μM TE/100g	7,92-15,62 μM TE/100g	1274-1539 μM TE/100g
R. idaeus var *Indian Summer* Freeze-dried berries [98]	MeOH (100%) + HCl (1%) maceration with ultrasound	~75 mg GAE/100g FW	*~750 μM TE/100g FW	~1.5 mM TE/100g FW	NA
R. idaeus var *Skeena* Freeze-dried berries [98]		~40 mg GAE/100g FW	*~850 μM TE/100g FW	~2 mM TE/100g FW	NA
R. idaeus var *Royal Purple* Freeze-dried berries [98]		~80 mg GAE/100g FW	*~800 μM TE/100g FW	~2.5 mM TE/100g FW	NA
R. idaeus var *Fallgold* Freeze-dried berries		43.2 mg GAE/100g FW	*~1000 μM TE/100g FW	~1 mM TE/100g FW	NA

[98]					
R. idaeus *9 different cultivar* Frozen berries [88]	EtOH (60%) maceration	115.7-182.97mg/100g FW	*~3100-4500 μM TE/100g FW	*~7000-9500 μM TE/100g FW	*~1000-1800 μM TE/100g FW
R. idaeus Lyophilized leaves [129]	EtOH (70%) refluxing extraction, partitioned with CH2Cl2	NA	IC50 92.0 μg/mL	(125 μg/mL) 268.0 μg/mL	IC50 6.40 μg/mL
R. idaeus Air-dried rhizomes [129]		NA	IC50 73.90 μg/mL	(125 μg/mL) 85.0 μg/mL	IC50 12.67 μg/mL
R. idaeus Lyophilized berries [129]		NA	IC50 79.1 μg/mL	(125 μg/mL) 100.8 μg/mL	IC50 43.0 μg/mL
R. idaeus Frozen berries [91]	MeOH (20,60, 100%) infusion (40, 60, 80°C) at different times (1, 8, 15 min) with ultrasound	383.0 ± 12 mg GAE/100g FW	*2900±110 μM TE/100g FW	NA	*3950±130 μM TE/100g FW
R. idaeus Fresh berries [130]	Homogenised and pressed	164.4 ± 5.1 mg GAE/100g FW	IC50 2.40±0.21 mg/ml	NA	NA
R. idaeus var *Polana* Frozen berries [99]	MeOH (80%) maceration	242.02-449.63 mg CAE/100g FW	40.22-50.71 %	*870-1288 μM TE/100g FW	NA
R. idaeus var *Polka* Frozen berries [99]		251.25-446.16 mg CAE/100g FW	29.72-44.21%	*867-1061 μM TE/100g FW	NA
R. idaeus var *Polesie* Frozen berries [99]		229.60-426.21 mg CAE/100g FW	31.34-41.23%	*796-910 μM TE/100g FW	NA
R. idaeus var *Poranna R.* Frozen berries [99]		182.58-406.02 mg CAE/100g FW	28.05-47.41%	*582-670 μM TE/100g FW	NA
R. idaeus var *Benefis* Frozen berries [99]		266.89-494.14 mg CAE/100g FW	42.17-48.54%	*947-951 μM TE/100g FW	NA
R. idaeus var *Laszka* Frozen berries [99]		220.16-549.02 mg CAE/100g FW	43.61-73.43%	*858-1555 μM TE/100g FW	NA
R. idaeus var *Radziejowa* Frozen berries [99]		266.59-358.31 mg CAE/100g FW	30-44.36%	*941-1015 μM TE/100g FW	NA
R. idaeus var *Sokolica* Frozen berries [99]		175.90-381.82 mg CAE/100g FW	33.92-42.38%	*660-803 μM TE/100g FW	NA
R. idaeus var *Willamette* Frozen berries [99]		310.60-320.39 mg CAE/100g FW	40.68-47.96%	*914-1089 μM TE/100g FW	NA
R. idaeus var *Bristol* Frozen berries [99]		541.87-1351.59 mg CAE/100g FW	55.35-61.41%	*2065-4405 μM TE/100g FW	NA

R. idaeus var *Litacz* Frozen berries [99]		582.98-1251.77 mg CAE/100g FW	56.89-66.28%	*2121-4390 µM TE/100g FW	NA
R. idaeus var *Brazos* Frozen berries [105]	Lyophilized	1,605±0.02 mg GAE/100g	NA	NA	NA
R. idaeus Berries [131]	Pomace pressed extraction, homogenization and MeOH (80%) maceration	*0.849±0.145 mg GAE/100mL	361.27±5.65 µM TE/100g	77.27±1.05 µM/100mL	NA
	Pomace pressed extraction, homogenization and MeOH (80%) + formic acid (1%) infusion with ultrasound	*2.779±0.125 mg GAE/100mL	567.00±4.56 µM TE/100g	100.27±1.22 µM/100mL	NA
R. idaeus var *Brillance* Freeze-dried pulp [132]	MeOH+water+formic acid (70:25:5) maceration	NA	IC50 205±10 µg/mL	NA	NA
R. idaeus Freeze-dried berries [133]	FM	NA	EC50 0.60 mg/mL	0.76 mM Fe2+/g	*108 mM TE/100g
	MeOH (80%) + formic acid (1%) maceration	NA		3.08 mM Fe2+/g	*438 mM TE/100g
R. idaeus var *Nova* Freeze-driad berries [134]	Acetone (50%) maceration	*6690 mg GAE/100g	*30.45 mM TE/100g DW	NA	NA
R. idaeus var *Dinkum* Freeze-driad berries [134]		*9850 mg GAE/100g	*35.13 mM TE/100g DW	NA	NA
R. idaeus var *Heritage* Freeze-driad berries [134]		*7920 mg GAE/100g	*32.6 mM TE/100g DW	NA	NA
R. idaeus var *Autumn Britten* Freeze-driad berries [134]		*7070 mg GAE/100g	*34.96 mM TE/100g DW	NA	NA
R. idaeus var *Josephine* Freeze-driad berries [134]		*8170 mg GAE/100g	34.89 mM TE/100g DW	NA	NA
R. idaeus var *Anne* Freeze-driad berries [134]		*4410 mg GAE/100g	*34.47 mM TE/100g DW	NA	NA
R. idaeus var *Fall Gold* Freeze-driad berries [134]		*4090 mg GAE/100g	*33.04 mM TE/100g DW	NA	NA
R. idaeus var *Cascade Delight* Fresh leaf buds [89]	Water maceration<br>EtOH (40%) maceration<br><br>Glycerol (40%) maceration	~18-44 mg GAE/100g FW	~9-18 mM TE/100g FW	NA	~5-12 mM TE/100g FW

R. idaeus var *Glen Fyne* Fresh leaf buds [89]	Water maceration<br>EtOH (40%) maceration<br>Glycerol (40%) maceration	~22-41 mg GAE/100g FW	~10-23 mM TE/100g FW	NA	~6-13 mM TE/100g FW
R. idaeus var *Octavia* Fresh leaf buds [89]	Water maceration<br>EtOH (40%) maceration<br>Glycerol (40%) maceration	30.85–47.45 mg GAE/100g FW	~16-24 mM TE/100g FW	NA	~10-12 mM TE/100g FW
R. idaeus var *Hybrid* Fresh leaf buds [89]	Water maceration<br>EtOH (40%) maceration<br>Glycerol (40%) maceration	~9-18 mg GAE/100g FW	~12-16 mM TE/100g FW	NA	~4-7 mM TE/100g FW
R. idaeus Freeze-dried berries [100]	MeOH (80%) + acetic acid (0,5%) maceration with sonication		[2 mg/mL] 46±0.5%	*9400±170 mg TE/100mL	[2 mg/mL] 23±0.2%
R. idaeus var *Chilliwack* Frozen berries [135]	EtOH(96%):water:glacial acetic acid(80:20:1) homogenization	325-363 mg GAE/100g	NA	41.4-42.2 μM FE/g	NA
R. idaeus var *Haida* Frozen berries [135]		352-375 mg GAE/100g	NA	39.4-44.8 μM FE/g	NA
R. idaeus var *Kohatu* Frozen berries [135]		291-296 mg GAE/100g	NA	34.8-38.2 μM FE/g	NA
R. idaeus var *Meeker* Frozen berries [135]		360-420 mg GAE/100g	NA	39-48.7 μM FE/g	NA
R. idaeus var *Qualicum* Frozen berries [135]		286-383 mg GAE/100g	NA	37.9-43.4 μM FE/g	NA
R. idaeus var *Tulameen* Frozen berries [135]		237-272 mg GAE/100g	NA	29.9-36.1 μM FE/g	NA
R. idaeus var *Citadel* Frozen berries [135]		269-271 mg GAE/100g	NA	32.7-33.5 μM FE/g	NA
R. idaeus var *F79* Frozen berries [135]		259-281 mg GAE/100g	NA	32.2-32.9 μM FE/g	NA
R. idaeus var *Kaituna* Frozen berries [135]		273-310 mg GAE/100g	NA	31.7-37.1 μM FE/g	NA
R. idaeus var *Summer* Frozen berries [135]		296-311 mg GAE/100g	NA	35.2-37.3 μM FE/g	NA
R. idaeus Berries [90]	MeOH (98%) + acetic acid (2%) maceration<br>Acetone (80%) maceration	*31 mg GAE/100g<br>*15 mg GAE/100g	*~48-68 μM TE/100g	NA<br>NA	*~290-460 μM TE/100g

References

1. FAO Crops and Livestock Products—Raspberries Production. License: CC BY-NC-SA 3.0 IGO. Available online: https://www.fao.org/faostat/en/#data/QCL (accessed on 14 April 2022).
2. Rao, A.V.; Snyder, D.M. Raspberries and human health: A review. *J. Agric. Food Chem.* **2010**, *58*, 3871–3883.
3. Ispiryan, A.; Viškelis, J. Valorisation of raspberries by-products for food and pharmaceutical industries. *Adv. Agric. Harti. and Ento. AAHE-102* **2019**.
4. Marić, B.; Pavlić, B.; Čolović, D.; Abramović, B.; Zeković, Z.; Bodroža-Solarov, M.; Ilić, N.; Teslić, N. Recovery of high-content ω–3 fatty acid oil from raspberry (*Rubus idaeus* L.) seeds: Chemical composition and functional quality. *LWT* **2020**, *130*, 109627.
5. Wang, L.; Lin, X.; Zhang, J.; Zhang, W.; Hu, X.; Li, W.; Li, C.; Liu, S. Extraction methods for the releasing of bound phenolics from *Rubus idaeus* L. leaves and seeds. *Ind. Crops Prod.* **2019**, *135*, 1–9.
6. Četojević-Simin, D.D.; Velićanski, A.S.; Cvetković, D.D.; Markov, S.L.; Ćetković, G.S.; Tumbas Šaponjac, V.T.; Vulić, J.J.; Čanadanović-Brunet, J.M.; Djilas, S.M. Bioactivity of Meeker and Willamette raspberry (*Rubus idaeus* L.) pomace extracts. *Food Chem.* **2015**, *166*, 407–413.
7. Chwil, M.; Kostryco, M. Bioactive compounds and antioxidant activity of *Rubus idaeus* L. Leaves. *Acta Sci. Pol. Hortorum Cultus* **2018**, *17*, 135–147.
8. Veljković, B.; Dordević, N.; Dolićanin, Z.; Ličina, B.; Topuzović, M.; Stanković, M.; Zlatić, N.; Dajić-Stevanović, Z. Antioxidant and anticancer properties of leaf and fruit extracts of the wild raspberry (*Rubus idaeus* L.). *Not. Bot. Horti Agrobot.* **2019**, *47*, 359–367.
9. Belščak-Cvitanović, A.; Komes, D.; Benković, M.; Karlović, S.; Hečimović, I.; Ježek, D.; Bauman, I. Innovative formulations of chocolates enriched with plant polyphenols from *Rubus idaeus* L. leaves and characterization of their physical, bioactive and sensory properties. *Food Res. Int.* **2012**, *48*, 820–830.
10. Costea, T.; Vlase, L.; Gostin, I.N.; Olah, N.K.; Predan, G.M.I. Botanical characterization, phytochemical analysis and antioxidant activity of indigenous red raspberry (*Rubus Idaeus* L.) leaves. *Stud. Univ. Vasile Goldis Arad Ser. Stiint. Vietii* **2016**, *26*, 463–472.
11. Durgo, K.; Belščak-Cvitanović, A.; Stančić, A.; Franekić, J.; Komes, D. The bioactive potential of red raspberry (*Rubus idaeus* L.) leaves in exhibiting cytotoxic and cytoprotective activity on human laryngeal carcinoma and colon adenocarcinoma. *J. Med. Food* **2012**, *15*, 258–268.
12. Ferlemi, A.V.; Lamari, F.N. Berry leaves: An alternative source of bioactive natural products of nutritional and medicinal value. *Antioxidants* **2016**, *5*, 17.
13. Ponder, A.; Hallmann, E. Phenolics and carotenoid contents in the leaves of different organic and conventional raspberry (*Rubus idaeus* l.) cultivars and their in vitro activity. *Antioxidants* **2019**, *8*, 458.
14. McCullough, A.R.; Parekh, S.; Rathbone, J.; Del Mar, C.B.; Hoffmann, T.C. A systematic review of the public's knowledge and beliefs about antibiotic resistance. *J. Antimicrob. Chemother.* **2016**, *71*, 27–33.
15. Holmes, A.H.; Moore, L.S.P.; Sundsfjord, A.; Steinbakk, M.; Regmi, S.; Karkey, A.; Guerin, P.J.; Piddock, L.J.V. Understanding the mechanisms and drivers of antimicrobial resistance. *Lancet* **2016**, *387*, 176–187.
16. Hashempour-Baltork, F.; Hosseini, H.; Shojaee-Aliabadi, S.; Torbati, M.; Alizadeh, A.M.; Alizadeh, M. Drug resistance and the prevention strategies in food borne bacteria: An update review. *Adv. Pharm. Bull.* **2019**, *9*, 335–347.
17. Acar, J.F.; Moulin, G. Antimicrobial resistance at farm level Resistant bacterial clones on the farm. *Rev. Sci. Tech. Off. Int. Epiz.* **2006**, *25*, 775–792.
18. Mensah, S.E.P.; Koudandé, O.D.; Sanders, P.; Laurentie, M.; Mensah, G.A.; Abiola, F.A. Antimicrobial residues in foods of animal origin in Africa: Public health risks. *Rev. Sci. Tech.* **2014**, *33*, 987–996.
19. Waterhouse, A.L. Determination of total phenolics. *Curr. Protoc. Food Anal. Chem.* **2002**, *6*, I1.1.1–I1.1.8.
20. Garzoli, S.; Laghezza Masci, V.; Franceschi, S.; Tiezzi, A.; Giacomello, P.; Ovidi, E. Headspace/GC–MS analysis and investigation of antibacterial, antioxidant and cytotoxic activity of essential oils and hydrolates from *Rosmarinus officinalis* L. and *Lavandula angustifolia* miller. *Foods* **2021**, *10*, 1768. https://doi.org/10.3390/foods10081768.
21. Rodolfi, M.; Chiancone, B.; Liberatore, C.M.; Fabbri, A.; Cirlini, M.; Ganino, T. Changes in chemical profile of Cascade hop cones according to the growing area. *J. Sci. Food Agric*. **2019**, *99*, 6011–6019.
22. Robertson, G.W.; Griffiths, D.W.; Woodford, J.A.T.; Birch, A.N.E. Changes in the chemical composition of volatiles released by the flowers and fruits of the red raspberry (*Rubus idaeus*) cultivar glen prosen. *Phytochemistry* **1995**, *38*, 1175–1179.
23. Adams, R.P. *Identification of Essential Oil Components by Gas Chromatography/Mass Spectroscopy*, 4th ed.; Allured Publishing Corporation: Carol Stream, IL, USA, 2007; Volume 456, pp. 544–545.
24. Yang, Y.N.; Zheng, F.P.; Yu, A.N.; Sun, B.G. Changes of the free and bound volatile compounds in *Rubus corchorifolius* L. f. fruit during ripening. *Food Chem.* **2019**, *287*, 232–240.
25. Nguyen, P. A. H.; Clark, E. R.; Ananthakrishnan, S.; Lenz, K.; Canavan, H. E. How to select the appropriate method (s) of cytotoxicity analysis of mammalian cells at biointerfaces: A tutorial. *Biointerphases*, **2020**, 15(3), 031201.
26. Hudzicki, J. Kirby-Bauer disk diffusion susceptibility test protocol author information. *Am. Soc. Microbiol.* **2009**, *15*, 1–13.
27. Blois, M.S. Antioxidant determinations by the use of a stable free radical. *Nature* **1958**, *181*, 1199–1200.

28. Bueno-Costa, F.M.; Zambiazi, R.C.; Bohmer, B.W.; Chaves, F.C.; Silva, W.P. da; Zanusso, J.T.; Dutra, I. Antibacterial and antioxidant activity of honeys from the state of Rio Grande do Sul, Brazil. *LWT Food Sci. Technol.* **2016**, *65*, 333–340.
29. Gül, A.; Pehlivan, T. Antioxidant activities of some monofloral honey types produced across Turkey. *Saudi J. Biol. Sci.* **2018**, *25*, 1056–1065.
30. Kratchanova, M.; Denev, P.; Ciz, M.; Lojek, A.; Mihailov, A. Evaluation of antioxidant activity of medicinal plants containing polyphenol compounds. Comparison of two extraction systems. *Acta Biochim. Pol.* **2010**, *57*, 229–234.
31. Pavlović, A.V.; Papetti, A.; Zagorac, D.Č.D.; Gašić, U.M.; Mišić, D.M.; Tešić, Ž.L.; Natić, M.M. Phenolics composition of leaf extracts of raspberry and blackberry cultivars grown in Serbia. *Ind. Crops Prod.* **2016**, *87*, 304–314.
32. Cai, Y.; Hu, X.; Huang, M.; Sun, F.; Yang, B.; He, J.; Wang, X.; Xia, P.; Chen, J. Characterization of the antibacterial activity and the chemical components of the volatile oil of the leaves of *Rubus parvifolius* L. *Molecules* **2012**, *17*, 7758–7768.
33. Li, Z.H.; Guo, H.; Xu, W. Bin; Ge, J.; Li, X.; Alimu, M.; He, D.J. Rapid identification of flavonoid constituents directly from PTP1B Inhibitive extract of raspberry (*Rubus idaeus* L.) leaves by HPLC-ESI-QTOF-MS-MS. *J. Chromatogr. Sci.* **2016**, *54*, 805–810.
34. Saad, N.; Louvet, F.; Tarrade, S.; Meudec, E.; Grenier, K.; Landolt, C.; Ouk, T.S.; Bressollier, P. Enzyme-assisted extraction of bioactive compounds from raspberry (*Rubus idaeus* L.) pomace. *J. Food Sci.* **2019**, *84*, 1371–1381.
35. Yang, J.; Cui, J.; Chen, J.; Yao, J.; Hao, Y.; Fan, Y.; Liu, Y. Evaluation of physicochemical properties in three raspberries (*Rubus idaeus*) at five ripening stages in northern China. *Sci. Hortic.* **2020**, *263*, 109146.
36. Koziol, A.; Stryjewska, A.; Librowski, T.; Salat, K.; Gawel, M.; Moniczewski, A.; Lochynski, S. An overview of the pharmacological properties and potential applications of natural monoterpenes. *Mini Rev. Med. Chem.* **2014**, *14*, 1156–1168.
37. De Sousa, J.P.; De Azerêdo, G.A.; De Araújo Torres, R.; Da Silva Vasconcelos, M.A.; Da Conceição, M.L.; De Souza, E.L. Synergies of carvacrol and 1,8-cineole to inhibit bacteria associated with minimally processed vegetables. *Int. J. Food Microbiol.* **2012**, *154*, 145–151.
38. Moteki, H.; Hibasami, H.; Yamada, Y.; Katsuzaki, H.; Imai, K.; Komiya, T. Specific induction of apoptosis by 1,8-cineole in two human leukemia cell lines, butnot a in human stomach cancer cell line. *Oncol. Rep.* **2002**, *9*, 757–760.
39. Murata, S.; Shiragami, R.; Kosugi, C.; Tezuka, T.; Yamazaki, M.; Hirano, A.; Yoshimura, Y.; Suzuki, M.; Shuto, K.; Ohkohchi, N.; et al. Antitumor effect of 1, 8-cineole against colon cancer. *Oncol. Rep.* **2013**, *30*, 2647–2652.
40. Kamatou, G.P.P.; Makunga, N.P.; Ramogola, W.P.N.; Viljoen, A.M. South African Salvia species: A review of biological activities and phytochemistry. *J. Ethnopharmacol.* **2008**, *119*, 664–672.
41. Khaleel, C.; Tabanca, N.; Buchbauer, G. α-Terpineol, a natural monoterpene: A review of its biological properties. *Open Chem.* **2018**, *16*, 349–361.
42. De Araújo-Filho, H.G.; dos Santos, J.F.; Carvalho, M.T.B.; Picot, L.; Fruitier-Arnaudin, I.; Groult, H.; Quintans-Júnior, L.J.; Quintans, J.S.S. Anticancer activity of limonene: A systematic review of target signaling pathways. *Phytoher. Res.* **2021**, *35*, 4957–4970.
43. Cai, R.; Hu, M.; Zhang, Y.; Niu, C.; Yue, T.; Yuan, Y.; Wang, Z. Antifungal activity and mechanism of citral, limonene and eugenol against *Zygosaccharomyces rouxii*. *LWT* **2019**, *106*, 50–56.
44. Kummer, R.; Fachini-Queiroz, F.C.; Estevão-Silva, C.F.; Grespan, R.; Silva, E.L.; Bersani-Amado, C.A.; Cuman, R.K.N. Evaluation of anti-inflammatory activity of citrus latifolia Tanaka essential oil and limonene in experimental mouse models. *Evid.-Based Complement. Altern. Med.* **2013**, *2013*, 859083.
45. Roberto, D.; Micucci, P.; Sebastian, T.; Graciela, F.; Anesini, C. Antioxidant activity of limonene on normal murine lymphocytes: Relation to H2O2 modulation and cell proliferation. *Basic Clin. Pharmacol. Toxicol.* **2009**, *106*, 38–44.
46. Zhu, Q.; Jiang, M.L.; Shao, F.; Ma, G.Q.; Shi, Q.; Liu, R.H. Chemical composition and antimicrobial activity of the essential oil from *Euphorbia helioscopia* L. *Nat. Prod. Commun.* **2020**, *15*, 549–555.
47. Attia, E.Z.; Abd El-Baky, R.M.; Desoukey, S.Y.; El Hakeem Mohamed, M.A.; Bishr, M.M.; Kamel, M.S. Chemical composition and antimicrobial activities of essential oils of *Ruta graveolens* plants treated with salicylic acid under drought stress conditions. *Futur. J. Pharm. Sci.* **2018**, *4*, 254–264.
48. Popova, A.A.; Koksharova, O.A.; Lipasova, V.A.; Zaitseva, J.V.; Katkova-Zhukotskaya, O.A.; Eremina, S.I.; Mironov, A.S.; Chernin, L.S.; Khmel, I.A. Inhibitory and toxic effects of volatiles emitted by strains of *Pseudomonas* and *Serratia* on growth and survival of selected microorganisms, *Caenorhabditis elegans*, and *Drosophila melanogaster*. *Biomed Res. Int.* **2014**, *11*, 125704.
49. Trombetta, D.; Saija, A.; Bisignano, G.; Arena, S.; Caruso, S.; Mazzanti, G.; Uccella, N.; Castelli, F. Study on the mechanisms of the antibacterial action of some plant α,β-unsaturated aldehydes. *Lett. Appl. Microbiol.* **2002**, *35*, 285–290.
50. Kamdem, S.S.; Belletti, N.; Magnani, R.; Lanciotti, R.; Gardini, F. Effects of carvacrol, (E)-2-hexenal, and citral on the thermal death kinetics of listeria monocytogenes. *J. Food Prot.* **2011**, *74*, 2070–2078.
51. Ma, W.; Zhao, L.; Zhao, W.; Xie, Y. (E)-2-Hexenal, as a potential natural antifungal compound, inhibits *Aspergillus flavus* spore germination by disrupting mitochondrial energy metabolism. *J. Agric. Food Chem.* **2019**, *67*, 1138–1145.
52. Ma, W.; Johnson, E.T. Natural flavour (E,E)-2,4-heptadienal as a potential fumigant for control of *Aspergillus flavus* in stored peanut seeds: Finding new antifungal agents based on preservative sorbic acid. *Food Control* **2021**, *124*, 107938.
53. Sartori, D.; Gaion, A. Toxicity of polyunsaturated aldehydes of diatoms to Indo-Pacific bioindicator organism *Echinometra mathaei*. *Drug Chem. Toxicol.* **2016**, *39*, 124–128.

54. Adaszyńska, M.; Swarcewicz, M.; Dzięciol, M.; Dobrowolska, A. Comparison of chemical composition and antibacterial activity of lavender varieties from Poland. *Nat. Prod. Res.* **2013**, *27*, 1497–1501.
55. Aprotosoaie, A.C.; Hăncianu, M.; Costache, I.I.; Miron, A. Linalool: A review on a key odorant molecule with valuable biological properties. *Flavour Fragr. J.* **2014**, *29*, 193–219.
56. Smith, R.L.; Waddell, W.J.; Cohen, S.M.; Feron, V.J.; Marnett, L.J.; Portoghese, P.S.; Rietjens, I.M.C.M.; Adams, T.B.; Gavin, C.L.; Mcgowen, M.M.; et al. GRAS flavoring substances 24. *Food Technol.* **2009**, *63*, 88.
57. Gadino, A.N.; Walton, V.M.; Lee, J.C. Evaluation of methyl salicylate lures on populations of *Typhlodromus pyri* (Acari: Phytoseiidae) and other natural enemies in western Oregon vineyards. *Biol. Control* **2012**, *63*, 48–55.
58. Kujur, A.; Yadav, A.; Kumar, A.; Singh, P.P.; Prakash, B. Nanoencapsulated methyl salicylate as a biorational alternative of synthetic antifungal and aflatoxin B1 suppressive agents. *Environ. Sci. Pollut. Res.* **2019**, *26*, 18440–18450.
59. Lu, X.P.; Liu, J.H.; Weng, H.; Ma, Z.Q.; Zhang, X. Efficacy of binary combinations between methyl salicylate and carvacrol against thrips *Anaphothrips obscurus*: Laboratory and field trials. *Pest Manag. Sci.* **2020**, *76*, 589–596.
60. Oloyede, G.K. Toxicity, antimicrobial and antioxidant activities of methyl salicylate dominated essential oils of *Laportea aestuans* (Gaud). *Arab. J. Chem.* **2016**, *9*, S840–S845.
61. Anzaku, A.A.; Akyala, J.I.; Juliet, A.; Obianuju, E.C. Antibacterial activity of lauric acid on some selected clinical isolates. *Ann. Clin. Lab. Res.* **2017**, *5*, 2.
62. Sado Kamdem, S.; Guerzoni, M.E.; Baranyi, J.; Pin, C. Effect of capric, lauric and α-linolenic acids on the division time distributions of single cells of *Staphylococcus aureus*. *Int. J. Food Microbiol.* **2008**, *128*, 122–128.
63. Shen, X.; Chen, W.; Zheng, Y.; Lei, X.; Tang, M.; Wang, H.; Song, F. Chemical composition, antibacterial and antioxidant activities of hydrosols from different parts of *Areca catechu* L. and *Cocos nucifera* L. *Ind. Crops Prod.* **2017**, *96*, 110–119.
64. Catalano, A.; Iacopetta, D.; Ceramella, J.; Scumaci, D.; Giuzio, F.; Saturnino, C.; Aquaro, S.; Rosano, C.; Sinicropi, M.S. Multidrug Resistance (MDR): A widespread phenomenon in pharmacological therapies. *Molecules* **2022**, *27*, 616.
65. Rodenak-Kladniew, B.; Castro, A.; Stärkel, P.; Galle, M.; Crespo, R. 1,8-Cineole promotes G0/G1 cell cycle arrest and oxidative stress-induced senescence in HepG2 cells and sensitizes cells to anti-senescence drugs. *Life Sci.* **2020**, *243*, 117271.
66. Sales, A.; Felipe, L. de O.; Bicas, J.L. Production, properties, and applications of α-terpineol. *Food Bioprocess Technol.* **2020**, *13*, 1261–1279.
67. Rodenak-Kladniew, B.; Castro, A.; Stärkel, P.; De Saeger, C.; García de Bravo, M.; Crespo, R. Linalool induces cell cycle arrest and apoptosis in HepG2 cells through oxidative stress generation and modulation of Ras/MAPK and Akt/mTOR pathways. *Life Sci.* **2018**, *199*, 48–59.
68. Maczka, W.; Winska, K.; Grabarczyk, M. One hundred faces of geraniol. *Molecules* **2020**, *25*, 3303.
69. Gu, X.; Yao, X.; Mei, J.; He, H.; Gao, X.; Du, Y.; Zhao, J.; Zhao, L.; Lai, X.; Shi, K. β-caryophyllene, a natural bicyclic sesquiterpene, induces apoptosis by inhibiting inflammation-associated proliferation in MOLT-4 leukemia cells. *Pharmacogn. Mag.* **2021**, *17*, 58.
70. Su, Y.C.; Hsu, K.P.; Wang, E.I.C.; Ho, C.L. Composition, in vitro cytotoxic, and antimicrobial activities of the flower essential oil of *Diospyros discolor* from Taiwan. *Nat. Prod. Commun.* **2015**, *10*, 1311–1314.
71. Wang, P.; Zhou, R.; Zhou, R.; Li, W.; Weerasinghe, J.; Chen, S.; Rehm, B.H.A.; Zhao, L.; Frentiu, F.D.; Zhang, Z.; et al. Cold atmospheric plasma for preventing infection of viruses that use ACE2 for entry. *Theranostics* **2022**, *12*, 2811–2832.
72. Zaręba, N.; Więcławik, K.; Kizek, R.; Hosnedlova, B.; Kepinska, M. The impact of fullerenes as doxorubicin nano-transporters on metallothionein and superoxide dismutase status in MCF-10A cells. *Pharmaceutics* **2022**, *14*, 102.
73. Abdalla, A.N.; Shaheen, U.; Abdallah, Q.M.A.; Flamini, G.; Bkhaitan, M.M.; Abdelhady, M.I.S.; Ascrizzi, R.; Bader, A. Proapoptotic activity of *Achillea membranacea* essential oil and its major constituent 1,8-cineole against A2780 ovarian cancer cells. *Molecules* **2020**, *25*, 1582.
74. Kohoude, M.J.; Gbaguidi, F.; Agbani, P.; Ayedoun, M.A.; Cazaux, S.; Bouajila, J. Chemical composition and biological activities of extracts and essential oil of *Boswellia dalzielii* leaves. *Pharm. Biol.* **2017**, *55*, 33–42.
75. Kumar, D.; Sukapaka, M.; Babu, G.D.K.; Padwad, Y. Chemical composition and in vitro cytotoxicity of essential oils from leaves and flowers of *Callistemon citrinus* from western himalayas. *PLoS ONE* **2015**, *10*, e0133823.
76. Sampath, S.; Veeramani, V.; Krishnakumar, G.S.; Sivalingam, U.; Madurai, S.L.; Chellan, R. Evaluation of in vitro anticancer activity of 1,8-Cineole–containing n-hexane extract of *Callistemon citrinus* (Curtis) Skeels plant and its apoptotic potential. *Biomed. Pharmacother.* **2017**, *93*, 296–307.
77. Yang, C.; Chen, H.; Chen, H.; Zhong, B.; Luo, X.; Chun, J. Antioxidant and anticancer activities of essential oil from gannan navel orange peel. *Molecules* **2017**, *22*, 1391.
78. Hassan, S.B.; Gali-Muhtasib, H.; Göransson, H.; Larsson, R. Alpha terpineol: A potential anticancer agent which acts through suppressing NF-κB signalling. *Anticancer Res.* **2010**, *30*, 1911–1919.
79. Brändle, G.; L'Huillier, A.G.; Wagner, N.; Gervaix, A.; Wildhaber, B.E.; Lacroix, L. First report of spontaneous peritonitis in a child. *BMC Infect. Dis.* **2014**, *14*, 719.
80. Pulcrano, G.; Balzaretti, M.; Grosini, A.; Piacentini, V.; Poddighe, D. First report of *Kocuria marina* bloodstream infection unrelated to a central venous catheter: A mini-review on an emerging and under-recognized opportunistic pathogen. *Infez. Med.* **2017**, *25*, 71–74.

81. De Paiva Anciens Ramos, G.L.; Vigoder, H.C.; dos Santos Nascimento, J. *Kocuria* spp. in foods: Biotechnological uses and risks for food safety. *Appl. Food Biotechnol.* **2021**, *8*, 79–88.
82. Thoma, R.; Seneghini, M.; Seiffert, S.N.; Vuichard Gysin, D.; Scanferla, G.; Haller, S.; Flury, D.; Boggian, K.; Kleger, G.-R.; Filipovic, M.; et al. The challenge of preventing and containing outbreaks of multidrug-resistant organisms and *Candida auris* during the coronavirus disease 2019 pandemic: Report of a carbapenem-resistant *Acinetobacter baumannii* outbreak and a systematic review of the literatu. *Antimicrob. Resist. Infect. Control* **2022**, *11*, 1–11.
83. Adewoyin, M.A.; Okoh, A.I. The natural environment as a reservoir of pathogenic and non-pathogenic *Acinetobacter* species. *Rev. Environ. Health* **2018**, *33*, 265–272.
84. Carvalheira, A.; Ferreira, V.; Silva, J.; Teixeira, P. Enrichment of *Acinetobacter* spp. from food samples. *Food Microbiol.* **2016**, *55*, 123–127.
85. Krauze-Baranowska, M.; Majdan, M.; Hałasa, R.; Głód, D.; Kula, M.; Fecka, I.; Orzeł, A. The antimicrobial activity of fruits from some cultivar varieties of *Rubus idaeus* and *Rubus occidentalis*. *Food Funct.* **2014**, *5*, 2536–2541.
86. Qin, Y.; Wang, L.; Liu, Y.; Zhang, Q.; Li, Y.; Wu, Z. Release of phenolics compounds from *Rubus idaeus* L. dried fruits and seeds during simulated in vitro digestion and their bio-activities. *J. Funct. Foods* **2018**, *46*, 57–65.
87. Sariburun, E.; Şahin, S.; Demir, C.; Türkben, C.; Uylaşer, V. Phenolic content and antioxidant activity of raspberry and blackberry cultivars. *J. Food Sci.* **2010**, *75*, C328–C335.
88. Lee, H.H.; Moon, Y.S.; Yun, H.K.; Park, P.J.; Kwak, E.J. Contents of bioactive constituents and antioxidant activities of cultivated and wild raspberries. *Korean J. Hortic. Sci. Technol.* **2014**, *32*, 115–122.
89. Krzepiłko, A.; Prażak, R.; Święciło, A. Chemical composition, antioxidant and antimicrobial activity of raspberry, blackberry and raspberry-blackberry hybrid leaf buds. *Molecules* **2021**, *26*, 327.
90. Guiné, R.P.F.; Soutinho, S.M.A.; Gonçalves, F.J. Phenolic compounds and antioxidant activity in red fruits produced in organic farming. *Croat. J. Food Sci. Technol* **2014**, *6*, 15–26.
91. Mihailović, N.R.; Mihailović, V.B.; Ćirić, A.R.; Srećković, N.Z.; Cvijović, M.R.; Joksović, L.G. Analysis of wild raspberries (*Rubus idaeus* L.): Optimization of the ultrasonic-assisted extraction of phenolics and a new insight in phenolics bioaccessibility. *Plant Foods Hum. Nutr.* **2019**, *74*, 399–404.
92. Dos Santos, S.S.; Paraíso, C.M.; Rodrigues, L.M.; Madrona, G.S. Agro-industrial waste as a source of bioactive compounds: Ultrasound-assisted extraction from blueberry (*Vaccinium myrtillus*) and raspberry (*Rubus idaeus*) pomace. *Acta Sci. Technol.* **2021**, *43*, 1–8.
93. Çekiç, Ç.; Özgen, M. Comparison of antioxidant capacity and phytochemical properties of wild and cultivated red raspberries (*Rubus idaeus* L.). *J. Food Compos. Anal.* **2010**, *23*, 540–544.
94. Tosun, M.; Ercisli, S.; Karlidag, H.; Sengul, M. Characterization of red raspberry (*Rubus idaeus* L.) genotypes for their physicochemical properties. *J. Food Sci.* **2009**, *74*, C575–C579.
95. Dudzinska, D.; Luzak, B.; Boncler, M.; Rywaniak, J.; Sosnowska, D.; Podsedek, A.; Watala, C. CD39/NTPDase-1 expression and activity in human umbilical vein endothelial cells are differentially regulated by leaf extracts from *Rubus caesius* and *Rubus idaeus*. *Cell. Mol. Biol. Lett.* **2014**, *19*, 361–380.
96. Grabek-Lejko, D.; Wojtowicz, K. Comparison of antibacterial and antioxidant properties of fruits and leaves of blackberry (*Rubus plicatus*) and raspberry (*Rubus idaeus*). *J. Microbiol. Biotechnol. Food Sci.* **2014**, *3*, 514–518.
97. Mîrza, A. Antioxidant actiyity of leaf and fruit extracts from *Rubus fruticosus*, *Rubus idaeus* and *Rubus loganobaccus* growing in the conditions of the Republic of Moldova. *Sci. Pap. Ser. Manag. Econ. Eng. Agric. Rural Dev.* **2021**, *21*, 363–372.
98. Toshima, S.; Hirano, T.; Kunitake, H. Comparison of anthocyanins, polyphenols, and antioxidant capacities among raspberry, blackberry, and Japanese wild *Rubus* species. *Sci. Hortic.* **2021**, *285*, 110204.
99. Kostecka-Gugała, A.; Ledwozyw-Smoleń, I.; Augustynowicz, J.; Wyzgolik, G.; Kruczek, M.; Kaszycki, P. Antioxidant properties of fruits of raspberry and blackberry grown in central Europe. *Open Chem.* **2015**, *13*, 1313–1325.
100. Ogawa, K.; Sakakibara, H.; Iwata, R.; Ishii, T.; Sato, T.; Goda, T.; Shimoi, K.; Kumazawa, S. Anthocyanin composition and antioxidant activity of the crowberry (*Empetrum nigrum*) and other berries. *J. Agric. Food Chem.* **2008**, *56*, 4457–4462.
101. Dvaranauskaite, A.; Venskutonis, P.R.; Labokas, J. Comparison of quercetin derivatives in ethanolic extracts of red raspberry (*Rubus idaeus* L.) leaves. *Acta Aliment.* **2008**, *37*, 449–461.
102. Venskutonis, P.R.; Dvaranauskaite, A.; Labokas, J. Radical scavenging activity and composition of raspberry (*Rubus idaeus*) leaves from different locations in Lithuania. *Fitoterapia* **2007**, *78*, 162–165.
103. Yu, R.; Chen, L.; Xin, X. Comparative assessment of chemical compositions, antioxidant and antimicrobial activity in ten berries grown in China. *Flavour Fragr. J.* **2020**, *35*, 197–208.
104. Gülçin, I.; Topal, F.; Çakmakçi, R.; Bilsel, M.; Gören, A.C.; Erdogan, U. Pomological features, nutritional quality, polyphenol content analysis, and antioxidant properties of domesticated and 3 wild ecotype forms of raspberries (*Rubus idaeus* L.). *J. Food Sci.* **2011**, *76*, 585–593.
105. Ramirez, M.R.; Apel, M.A.; Raseira, M.C.B.; Zuanazzi, J.Â.S.; Henriques, A.T. Polyphenol content and evaluation of antichemotactic, antiedematogenic and antioxidant activities of *Rubus* sp. cultivars. *J. Food Biochem.* **2011**, *35*, 1389–1397.
106. Değirmenci, H.; Erkurt, H. Relationship between volatile components, antimicrobial and antioxidant properties of the essential oil, hydrosol and extracts of *Citrus aurantium* L. flowers. *J. Infect. Public Health* **2020**, *13*, 58–67.

107. Flores-Soto, M.E.; Corona-Angeles, J.A.; Tejeda-Martinez, A.R.; Flores-Guzman, P.A.; Luna-Mujica, I.; Chaparro-Huerta, V.; Viveros-Paredes, J.M. β-Caryophyllene exerts protective antioxidant effects through the activation of NQO1 in the MPTP model of Parkinson's disease. *Neurosci. Lett.* **2021**, *742*, 135534.
108. Lin, L.; Long, N.; Qiu, M.; Liu, Y.; Sun, F.; Dai, M. The inhibitory efficiencies of geraniol as an anti-inflammatory, antioxidant, and antibacterial, natural agent against methicillin-resistant *Staphylococcus aureus* infection in vivo. *Infect. Drug Resist.* **2021**, *14*, 2991–3000.
109. Taheri Mirghaed, A.; Fayaz, S.; Hoseini, S.M. Effects of dietary 1,8-cineole supplementation on serum stress and antioxidant markers of common carp (*Cyprinus carpio*) acutely exposed to ambient ammonia. *Aquaculture* **2019**, *509*, 8–15.
110. Zheljazkov, V.D.; Kacaniova, M.; Dincheva, I.; Radoukova, T.; Semerdjieva, I.B.; Astatkie, T.; Schlegel, V. Essential oil composition, antioxidant and antimicrobial activity of the galbuli of six juniper species. *Ind. Crops Prod.* **2018**, *124*, 449–458.
111. Szymanowska, U.; Baraniak, B.; Bogucka-Kocka, A. Antioxidant, Anti-Inflammatory, and Postulated Cytotoxic Activity of Phenolic and Anthocyanin-Rich Fractions from Polana Raspberry (*Rubus idaeus* L.) Fruit and Juice-In Vitro Study. *Molecules* **2018**, *23*, 1812.
112. Benvenuti, S.; Pellati, F.; Melegari, M.; Bertelli, D. Polyphenols, anthocyanins, ascorbic acid, and radical scavenging activity of rubus, ribes, and aronia. *J. Food Sci.* **2004**, *69*, 164–169.
113. Dvaranauskaite, A.; Venskutonis, P.R.; Labokas, J. Radical scavenging activity of raspberry (*Rubus idaeus* L.) fruit extracts. *Acta Aliment.* **2006**, *35*, 73–83.
114. Buřičová, L.; Andjelkovic, M.; Čermáková, A.; Réblová, Z.; Jurček, O.; Kolehmainen, E.; Verhé, R.; Kvasnička, F. Antioxidant capacity and antioxidants of strawberry, blackberry, and raspberry leaves. *Czech J. Food Sci.* **2011**, *29*, 181–189.
115. Frías-Moreno, M.N.; Parra-Quezada, R.Á.; Ruíz-Carrizales, J.; González-Aguilar, G.A.; Sepulveda, D.; Molina-Corral, F.J.; Jacobo-Cuellar, J.L.; Olivas, G.I. Quality, bioactive compounds and antioxidant capacity of raspberries cultivated in northern Mexico. *Int. J. Food Prop.* **2021**, *24*, 603–614.
116. Costea, T.; Lupu, A.R.; Vlase, L.; Nencu, I.; Gîrd, C.E. Phenolic content and antioxidant activity of a raspberry leaf dry extract. *Rom. Biotechnol. Lett.* **2016**, *21*, 11346–11356.
117. Gramza-Michałowska, A.; Bueschke, M.; Kulczyński, B.; Gliszczyńska-Świgło, A.; Kmiecik, D.; Bilska, A.; Purłan, M.; Wałęsa, L.; Ostrowski, M.; Filipczuk, M.; et al. Phenolic compounds and multivariate analysis of antiradical properties of red fruits. *J. Food Meas. Charact.* **2019**, *13*, 1739–1747.
118. Frías-Moreno, M.N.; Parra-Quezada, R.A.; González-Aguilar, G.; Ruíz-Canizales, J.; Molina-Corral, F.J.; Sepulveda, D.R.; Salas-Salazar, N.; Olivas, G.I. Quality, bioactive compounds, antioxidant capacity, and enzymes of raspberries at different maturity stages, effects of organic vs. Conventional fertilization. *Foods* **2021**, *10*, 953.
119. Gao, W.; Wang, Y. shuai; Hwang, E.; Lin, P.; Bae, J.; Seo, S.A.; Yan, Z.; Yi, T.H. *Rubus idaeus* L. (red raspberry) blocks UVB-induced MMP production and promotes type I procollagen synthesis via inhibition of MAPK/AP-1, NF-κβ and stimulation of TGF-β/Smad, Nrf2 in normal human dermal fibroblasts. *J. Photochem. Photobiol. B Biol.* **2018**, *185*, 241–253.
120. Kafkas, E.; Ozgen, M.; Ozogul, Y.; Turemis, N. Phytochemical and Fatty Acid Profile of Selected Red Raspberry cultivars: A comparative study. *J. Food Qual.* **2008**, *31*, 67–78.
121. Wu, L.; Liu, Y.; Qin, Y.; Wang, L.; Wu, Z. HPLC-ESI-qTOF-MS/MS characterization, antioxidant activities and inhibitory ability of digestive enzymes with molecular docking analysis of various parts of raspberry (*Rubus ideaus* L.). *Antioxidants* **2019**, *8*, 274.
122. Ozarda, O.; Barla Demirkoz, A.; Özdemir, M. Sensory characteristics and antioxidant capacity of red raspberry extract as a preservative in fruity flavoured beverages. *J. Food Sci. Technol.* **2015**, *52*, 6687–6694.
123. Radočaj, O.; Vujasinović, V.; Dimić, E.; Basić, Z. Blackberry (*Rubus fruticosus* L.) and raspberry (*Rubus idaeus* L.) seed oils extracted from dried press pomace after longterm frozen storage of berries can be used as functional food ingredients. *Eur. J. Lipid Sci. Technol.* **2014**, *116*, 1015–1024.
124. Parry, J.; Su, L.; Luther, M.; Zhou, K.; Peter Yurawecz, M.; Whittaker, P.; Yu, L. Fatty acid composition and antioxidant properties of cold-pressed marionberry, boysenberry, red raspberry, and blueberry seed oils. *J. Agric. Food Chem.* **2005**, *53*, 566–573.
125. Golmohamadi, A.; Möller, G.; Powers, J.; Nindo, C. Effect of ultrasound frequency on antioxidant activity, total phenolic and anthocyanin content of red raspberry puree. *Ultrason. Sonochem.* **2013**, *20*, 1316–1323.
126. Miletic, N.; Leposavic, A.; Popovic, B.; Mitrovic, O.; Kandic, M. Chemical and antioxidant properties of fully matured raspberry fruits (*Rubus idaeus* l.) picked in different moments of harvesting season. *Acta Hortic.* **2015**, *1099*, 211–218.
127. Marhuenda, J.; Alemán, M.D.; Gironés-vilaplana, A.; Pérez, A.; Caravaca, G.; Figueroa, F.; Mulero, J.; Zafrilla, P. Phenolic composition, antioxidant activity, and in vitro availability of four different berries. *J. Chem.* **2016**, *7*, 5194901.
128. Farias-Cervantes, V.S.; Chávez-Rodríguez, A.; García-Salcedo, P.A.; García-López, P.M.; Casas-Solís, J.; Andrade-González, I. Antimicrobial effect and in vitro release of anthocyanins from berries and Roselle obtained via microencapsulation by spray drying. *J. Food Process. Preserv.* **2018**, *42*, 1–8.
129. Xu, Y.; Li, L.Z.; Cong, Q.; Wang, W.; Qi, X.L.; Peng, Y.; Song, S.J. Bioactive lignans and flavones with in vitro antioxidant and neuroprotective properties from *Rubus idaeus* rhizome. *J. Funct. Foods* **2017**, *32*, 160–169.
130. Konić-Ristić, A.; Šavikin, K.; Zdunić, G.; Janković, T.; Juranic, Z.; Menković, N.; Stanković, I. Biological activity and chemical composition of different berry juices. *Food Chem.* **2011**, *125*, 1412–1417.

131. Krivokapić, S.; Vlaović, M.; Vratnica, B.D.; Perović, A.; Perovic, S. Biowaste as a potential source of bioactive compound-a case study of raspberry fruit pomace. *Foods* **2021**, *10*, 706.
132. Malone, N. *Strawberries—Cultivation, Antioxidant Properties and Health Benefits*; Nova Publishers: New York, NY, USA, 2014. ISBN 9781631172557.
133. Zorzi, M.; Gai, F.; Medana, C.; Aigotti, R.; Morello, S.; Peiretti, P.G. Bioactive compounds and antioxidant capacity of small berries. *Foods* **2020**, *9*, 623.
134. Zhang, L.; Li, J.; Hogan, S.; Chung, H.; Welbaum, G.E.; Zhou, K. Inhibitory effect of raspberries on starch digestive enzyme and their antioxidant properties and phenolic composition. *Food Chem.* **2010**, *119*, 592–599.
135. Connor, A.M.; Stephens, M.J.; Hall, H.K.; Alspach, P.A. Variation and heritabilities of antioxidant activity and total phenolic content estimated from a red raspberry factorial experiment. *J. Am. Soc. Hortic. Sci.* **2005**, *130*, 40.
